# Occupational-related risk of testing SARS-CoV-2 positive for publicly employed medical doctors in Sweden: A nationwide cohort study

**DOI:** 10.1177/14034948241304487

**Published:** 2024-12-26

**Authors:** Osvaldo Fonseca-Rodriguez, Emma Tobjörk, Hanna Jerndal, Marie Eriksson, Anne-Marie Fors Connolly

**Affiliations:** 1 Department of Clinical Microbiology, Umeå University, Sweden; 2 Department of Statistics, USBE, Umeå University, Sweden

**Keywords:** Occupational health, healthcare workers, COVID-19, SARS-CoV-2, risk factors, medical doctors

## Abstract

**Aims:**

Doctors have an increased risk of SARS-CoV-2 infection caused by exposure to contagious patients. We aimed to identify which clinical specialities among medical doctors had the highest occupation-related risk of testing positive for SARS-CoV-2, utilizing data for all publicly employed medical doctors in Sweden.

**Methods:**

Data regarding positive SARS-CoV-2 test results and employment for publicly employed doctors in Sweden were divided into three observation periods: 1) 1 February to 31 December 2020, 2) 1 January to 30 June 2021 and 3) 1 July 2021 to 31 March 2022. Individuals were stratified according to occupation clinic and compared with clinical occupations with little to no patient contact. The risk of testing positive for SARS-CoV-2 was estimated using Cox proportional hazards regression, with sex, age and vaccination status as covariates.

**Results:**

The study cohort included all publicly employed doctors in Sweden: 35,028 individuals. In the first period, Infectious Disease doctors had the highest incidence of SARS-CoV-2 positive tests, with an incidence of 20.2 %, compared with 8.7 % in the reference group, and an adjusted hazard ratio of 2.5 (95% confidence interval 2.02–3.04), which decreased during period 2–3. Doctors in Geriatric Medicine had an elevated risk throughout the whole study period.

**Conclusions:**

**Our study shows an association between working in a speciality that involves caring for contagious COVID-19 patients, which raises concerns about infection control measures and routines being insufficient to prevent occupational infection in future pandemics.**

## Introduction

The Coronavirus Disease 2019 (COVID-19) caused by the severe acute respiratory syndrome coronavirus 2 (SARS-CoV-2) emerged at the end of 2019 as an outbreak in China, subsequently spread rapidly across the world and was declared a pandemic by the World Health Organization in March 2020 (Nature 2022) [1]. The pandemic followed the same epidemiological pattern in Sweden as in other parts of Europe, with a first wave in spring/early summer 2020, a second wave starting in autumn 2021, closely followed by a third wave lasting until summer 2022, and a fourth wave during winter/spring 2022 [2]. Vaccination of medical staff against SARS-CoV-2 was rolled out in Sweden during late 2020.

SARS-CoV-2 infection can be asymptomatic or cause only mild respiratory symptoms, but can also result in severe pneumonitis causing critical illness, and in addition the virus can also affect other organ systems than the respiratory, as well as give rise to thromboembolic events [3,4]. SARS-CoV-2 is transmitted mainly via contact or respiratory droplets, but aerosol transmission is of significance in indoor spaces, as well as in the case of healthcare workers (HCWs) performing aerosol producing medical procedures such as tracheal intubation, non-invasive ventilation, tracheotomy and manual ventilation before intubation [5,6].

In Sweden, a nationwide study by the Swedish Public Health authorities showed that HCWs had a high incidence of COVID-19 compared with teachers and other professions with customer contact during the first and part of the second pandemic wave [7]. Though HCW is an overarching category, it comprises many different professions with differing work tasks and various amounts of exposure to COVID-19 patients, making it difficult to assess the impact on doctors. Previous studies show that HCWs have an additional risk of exposure to SARS-CoV-2 compared with the general population, through contact with contagious patients [8–13]. In an effort to pinpoint the occupational SARS-CoV-2 infection risk, some studies, covering the first year of the pandemic, have focused on severe COVID-19 rather than risk of SARS-CoV-2 infection [9–13]. However, hospitalization or fatality due to COVID-19 are relatively rare, therefore measuring these outcomes does not give a true picture of the risk of occupation-related infection with SARS-CoV-2, especially in the post-vaccination era when severe manifestations of COVID-19 have decreased drastically.

We aimed to identify which clinical specialities among medical doctors had the highest occupation-related risk of testing positive for SARS-CoV-2, utilizing unique data for all publicly employed medical doctors in Sweden administered by the Sweden Association of Local Authorities and Regions. According to the National Board of Health and Welfare (NBHW), publicly employed doctors correspond to approximately 75% of all medical doctors working in Sweden.

## Methods

### Study population

The study population comprised all publicly employed doctors in Sweden between 1 February 2020 and 31 March 2022. The majority of hospitals in Sweden are publicly owned, thereby data for all publicly employed doctors in these professions comprises the majority of hospital-based clinical doctors in Sweden [14]. Data from the salary database of the Swedish Municipalities and Councils was utilized to specify which speciality clinic the doctor was employed in regardless of career stage (Supplemental material Table SI online). The database exists only for doctors, precluding the inclusion of other categories of HCWs.

### Positive laboratory-verified SARS-CoV-2 test

The outcome variable of our study was having a registered positive test for SARS-CoV-2. This variable was retrieved from the communicable disease surveillance system SmiNet administered by the Public Health Agency of Sweden [15]. SmiNet is an electronic system allowing laboratories and practising doctors to report in real-time positive tests for communicable diseases that are notifiable by Swedish law. SARS-CoV-2 positive tests are obligatory to report to SmiNet; however, negative test results are not included [15]. The COVID-19 index date was based on the earliest registered of the following dates that were registered in the SmiNet database (if present) from the date of disease onset, to the sample date to date of diagnosis, and if these dates were unavailable, then the date of report to SmiNet was chosen. In cases where one individual had multiple registered positive tests, a positive result that occurred after a minimum of six months had elapsed since the previous positive test was considered a new infection.

### Definition of clinical speciality as the primary exposure variable

The primary exposure variable was the clinical speciality in which the doctors worked, and was stratified into the following categories (Supplemental Table I): General Practice; Infectious Diseases; Emergency Medicine; Anaesthesiology and Intensive Care; Internal Medicine (including Cardiology, Medical Gastroenterology and Hepatology, Endocrinology and Diabetology, Nephrology, Pulmonary Medicine, Allergology, and Internal Medicine); Geriatric Medicine; Neurological and Neurosurgical specialties (Neurology, Neurosurgery, Clinical Neurophysiology, and Rehabilitation Medicine); Surgery and Orthopaedics (including General Surgery, Thoracic Surgery, Orthopaedics, Obstetrics and Gynaecology, Gynaecological Oncology, Paediatric Surgery, Hand Surgery, Vascular Surgery, Plastic Surgery, and Urology); Ophthalmology and Ear–Nose–Throat Medicine (ENT); Paediatrics (including Paediatric Medicine, Paediatric Allergology, Paediatric Neurology and Rehabilitation, Neonatology, Paediatric Haematology and Oncology, and Paediatric Cardiology), Paediatric Psychiatry and School Health Care Medicine; Psychiatry (including General Psychiatry, Forensic Psychiatry, Addiction Medicine, and Geriatric Psychiatry), Oncology and Haematology; Dermatology, Palliative- and Pain Medicine, and Rheumatology.

The following clinical specialities were considered having little to no patient contact and constituted the reference group: Clinical Immunology and Transfusion Medicine, Clinical Microbiology, Infection Control, Clinical Chemistry, Pathology, Forensic Pathology, Clinical Pharmacology, Clinical Genetics, Occupational Medicine, Social Medicine (Public Health), Radiology, Neuroradiology, Nuclear Medicine, and Clinical Physiology.

Physicians with an unknown speciality were excluded from the analysis (Supplemental Figure 1 online).

### Statistical analyses

The observation period was divided into three periods: 1) 1 February to 31 December 2020; 2) 1 January to 30 June 2021; 3) 1 July 2021 to 31 March 2022. These time periods represent the pre-vaccine period, the vaccine roll out period and the post-vaccine time periods, respectively. The frequency distribution was described for categorical variables (e.g. SARS-CoV-2 infection, clinical speciality, sex, and vaccination status), along with mean and standard deviation for age as a continuous variable. Data regarding COVID-19 vaccination was derived from the National Vaccination Registry administered by the Swedish Public Health Agency. Individuals were considered not vaccinated until two weeks after the date they received their first vaccine dose. Individuals who died were censored at the death date.

To explore the association between positive SARS-CoV-2 test and working in a clinical speciality, along with additional explanatory variables, Cox proportional hazards regression analysis was performed. Vaccination was included as a time-dependent covariate, to account for changes in individuals’ vaccination status over time. Age and sex were also included as covariates. Hazard ratios and 95% confidence intervals (CIs) were estimated in univariable and multivariable (adjusted hazard ratio (aHR)) models for each period. Proportional hazard assumptions were assessed graphically using Schoenfeld residual plots for each subperiod. All analyses were conducted using the statistical software R version 4.1.1.

### Ethics approval

This study is an observational study and received ethical approval by the Swedish Ethical Review Board (No. 2020-02150).

## Results

A total of 35,028 individual doctors were included in our study, with 32,098 doctors included during February–December 2020 (period 1) and 32,654 doctors included during January 2021 to March 2022, which was further divided into period 2 (January–June 2021) and period 3 (July 2021 to March 2022) (Supplemental Figure S1). The average age of the cohort was 45 years, with approximately half being women (Table I). Vaccination coverage increased from 0.3% in period 1 to 97% in period 3 (Table I).

**Table I.** Overview of characteristics of doctors during the three studied time periods.

	Feb 2020 to Dec 2020 *N* = 32,098	Jan 2021 to June 2021 *N* = 32,654	Jul 2021 to Mar 2022 *N* = 32,654
**COVID-19**			
Not positive	28,180 (87.79%)	30,955 (94.80%)	24,673 (75.56%)
Positive	3918 (12.21%)	1699 (5.20%)	7981 (24.44%)
**Clinical speciality**			
Specialities with little to no patient contact	3063 (9.54%)	3143 (9.63%)	3143 (9.63%)
Anaesthesiology and Intensive Care	2132 (6.64%)	2189 (6.70%)	2189 (6.70%)
Dermatology and Venereal Diseases	400 (1.25%)	423 (1.30%)	423 (1.30%)
Ear–Nose–Throat	718 (2.24%)	724 (2.22%)	724 (2.22%)
Emergency Medicine	707 (2.20%)	745 (2.28%)	745 (2.28%)
General Practitioners	5857 (18.25%)	5929 (18.16%)	5929 (18.16%)
Geriatrics	571 (1.78%)	586 (1.79%)	586 (1.79%)
Infectious Diseases	718 (2.24%)	721 (2.21%)	721 (2.21%)
Internal Medicine	4288 (13.36%)	4351 (13.32%)	4351 (13.32%)
Neurology	978 (3.05%)	1016 (3.11%)	1016 (3.11%)
Oncology and Haematology	999 (3.11%)	1027 (3.15%)	1027 (3.15%)
Ophthalmology	761 (2.37%)	780 (2.39%)	780 (2.39%)
Paediatric Psychiatry, School Health Care	496 (1.55%)	507 (1.55%)	507 (1.55%)
Paediatrics	1939 (6.04%)	1984 (6.08%)	1984 (6.08%)
Palliative and Pain Medicine	56 (0.17%)	78 (0.24%)	78 (0.24%)
Psychiatry	2156 (6.72%)	2175 (6.66%)	2175 (6.66%)
Rheumatology	332 (1.03%)	340 (1.04%)	340 (1.04%)
Surgery and Orthopaedics	5927 (18.47%)	5936 (18.18%)	5936 (18.18%)
**Career stage**			
Attendings	17,741 (55.27%)	18,267 (55.94%)	18,267 (55.94%)
Attendings-GP	3430 (10.69%)	3530 (10.81%)	3530 (10.81%)
Residents	10,611 (33.06%)	10,541 (32.28%)	10,541 (32.28%)
Interns	316 (0.98%)	316 (0.97%)	316 (0.97%)
**Sex**			
Women	16,605 (51.73%)	16,985 (52.02%)	16,985 (52.02%)
Men	15,493 (48.27%)	15,669 (47.98%)	15,669 (47.98%)
**Age, years**	45.49 (11.83)	45.66 (11.97)	45.66 (11.97)
**Vaccination**			
Unvaccinated	32,019 (99.75%)	3716 (11.38%)	977 (2.99%)
Vaccinated	79 (0.25%)	28,938 (88.62%)	31,677 (97.01%)

Values are *n* (%) or mean (SD).

GP: General Practitioner

### Working in an Infectious Disease clinic carried the highest risk of testing PCR positive for SARS-CoV-2 for doctors during February–December 2020

**Figure 1.**
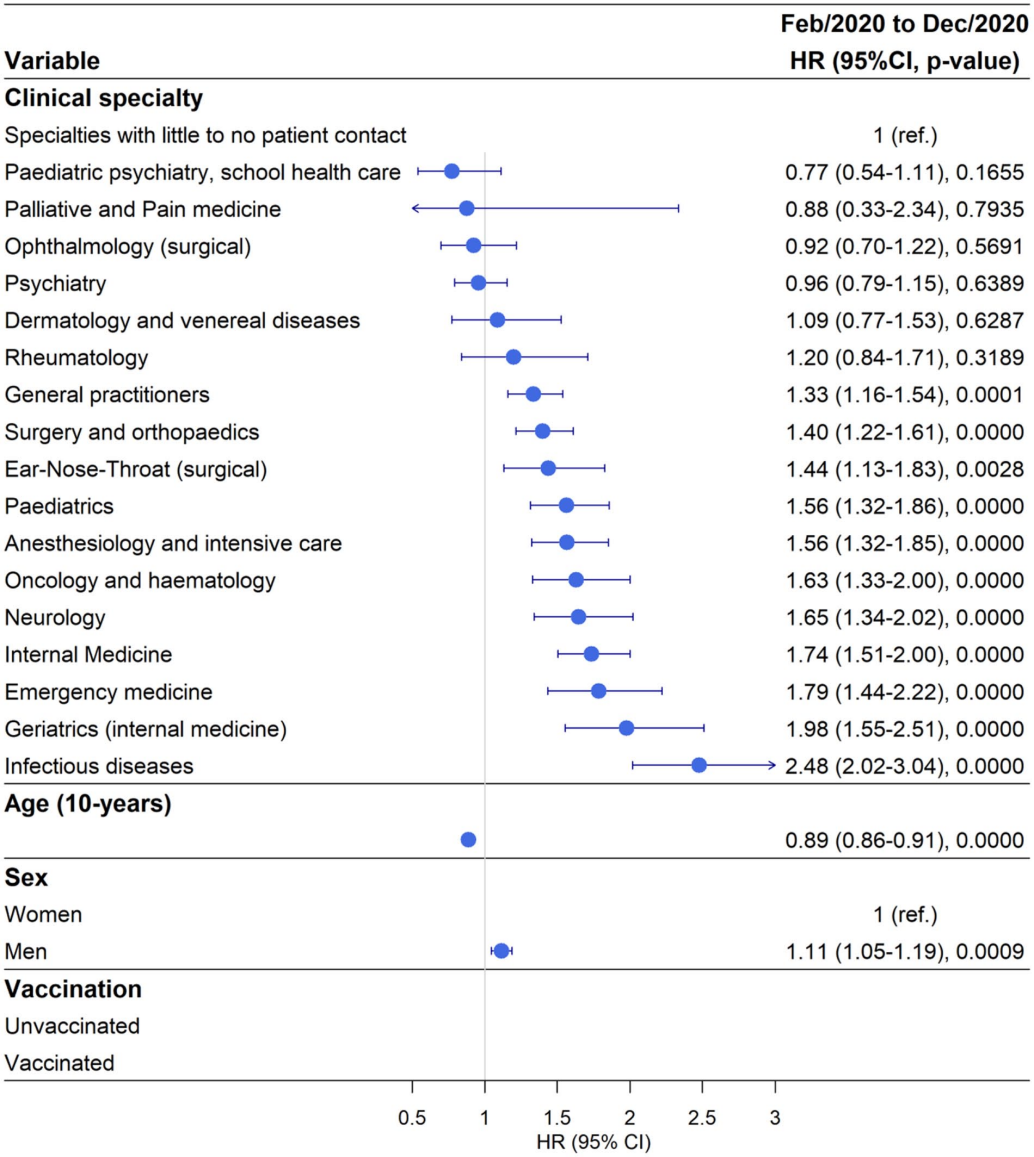
Risk of testing positive for SARS-CoV-2 by clinical speciality adjusted for age and sex during the time period of February–December 2020. HR: hazard ratio; CI: confidence interval; ref.: reference

Out of 32,098 doctors a total of 3918 tested positive for SARS-CoV-2 (12.2 %) during February–December 2020 (period 1) and in the reference group 8.72% tested positive (Table I). During this period, 20% of doctors working in Infectious Disease clinics tested positive for SARS-CoV-2, with aHR 2.48, 95% CI 2.02–3.04 compared with the reference group (Figure 1 and Supplemental Tables SII and SIII). This was followed by 16% of doctors working in Geriatric Medicine clinics testing positive for SARS-CoV-2 with aHR 1.98, 95% CI 1.55–2.51, and 17% of doctors working in Emergency Medicine clinics testing SARS-CoV-2 positive with aHR 1.79, 95% CI 1.44–2.22. There were 15% of doctors working in Internal Medicine clinics that tested positive for SARS-CoV-2 with aHR 1.74, 95% CI 1.51–2 (Figure 1 and Supplemental Tables SII and SIII). Doctors working in several other hospital-based clinics (Neurology and Neurosurgery, Oncology and Haematology, Anaesthesiology and Intensive Care, Paediatrics, ENT, Surgery and Orthopaedics) as well as General Practitioners (GPs) also had a significantly elevated risk of testing positive for SARS-CoV-2 compared with the reference group. Generally, the results were similar in the univariable model and the multivariable model adjusting for age and sex (Figure 1 and Supplemental Tables SII and SIII). For doctors working in the following clinics there was no significantly increased risk of testing SARS-CoV-2 PCR (polymerase chain reaction) positive: Rheumatology, Dermatology, Psychiatry, Ophthalmology, Palliative and Pain Medicine, and Paediatric Psychiatry and School Health Care Medicine compared with the reference group (Figure 1 and Supplemental Tables SII and SIII). Men had an 11% higher risk of testing positive for SARS-CoV-2 compared with women. For every 10-year increment in age, the risk of testing positive decreased by 11%. During this time period very few doctors were vaccinated, therefore this was not included in the model.

### Working in Paediatric Psychiatry and School Health Care carried the highest risk of testing PCR positive for SARS-CoV-2 for doctors during January–June 2021

**Figure 2.**
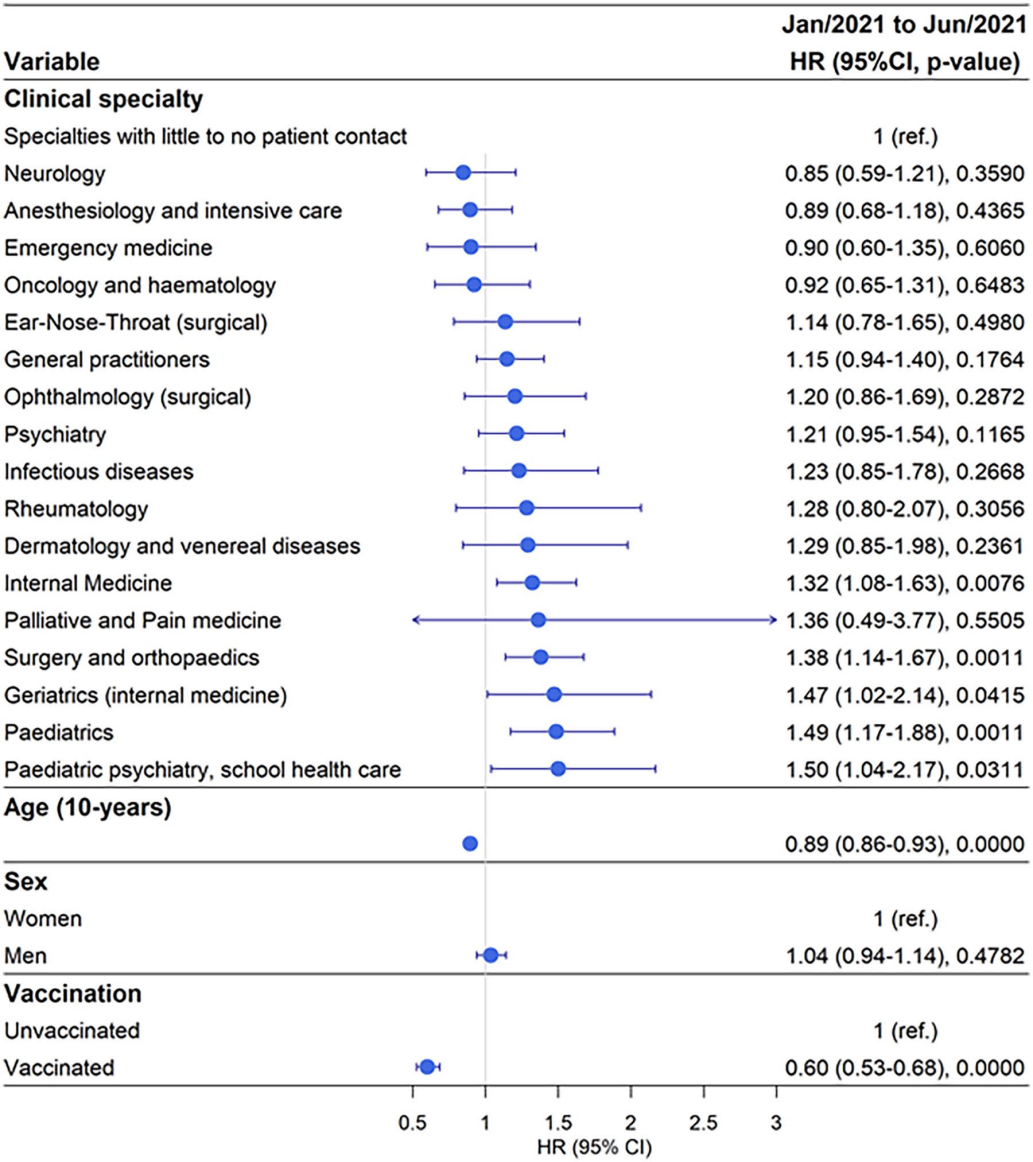
Risk of testing positive for SARS-CoV-2 by clinical speciality adjusted for age, sex and vaccination status during the time period January–June 2021. HR: hazard ratio; CI: confidence interval; ref.: reference

Out of 32,654 doctors a total of 1699 (5.2 %) tested positive for SARS-CoV-2 during period 2, January–June 2021, with 4.61% testing positive in the reference category (Table I and Supplemental Table SIV). During this period, 7% of doctors working in Paediatric Psychiatry and School Health Care tested positive for SARS-CoV-2 with aHR 1.5 95 %, CI 1.04–2.17 compared with the reference group (Figure 2 and Supplemental Tables SIV and SV). A similar proportion of doctors working in paediatric clinics (6.5%) tested positive for SARS-CoV-2 with aHR 1.49, 95% CI 1.17–1.88, followed by doctors working in geriatric clinics with 6% testing positive and aHR 1.47, 95% CI 1.02–2.14 compared with the reference group.

A similar risk profile existed for doctors working in Surgery and Orthopaedic clinics and Internal Medicine clinics with approximately 6% testing SARS-CoV-2 positive and approximately 1.3-fold increased risk (Figure 2 and Supplemental Tables SIV and SV). Doctors working in other clinics did not have a significantly increased risk of testing SARS-CoV-2 positive compared with the reference clinical specialities. In this period, men and women had a similar risk for testing positive; however, increasing age reduced the risk of testing SARS-CoV-2 positive with 11% for every 10-year increment (Figure 2 and Supplemental Tables SIV and SV). The effects of vaccination were evident, with vaccinated doctors showing a 40% reduced risk of testing positive for SARS-CoV-2 compared with unvaccinated doctors.

### Working in Paediatrics carried the highest risk of testing PCR positive for SARS-CoV-2 during July 2021 to March 2022

**Figure 3.**
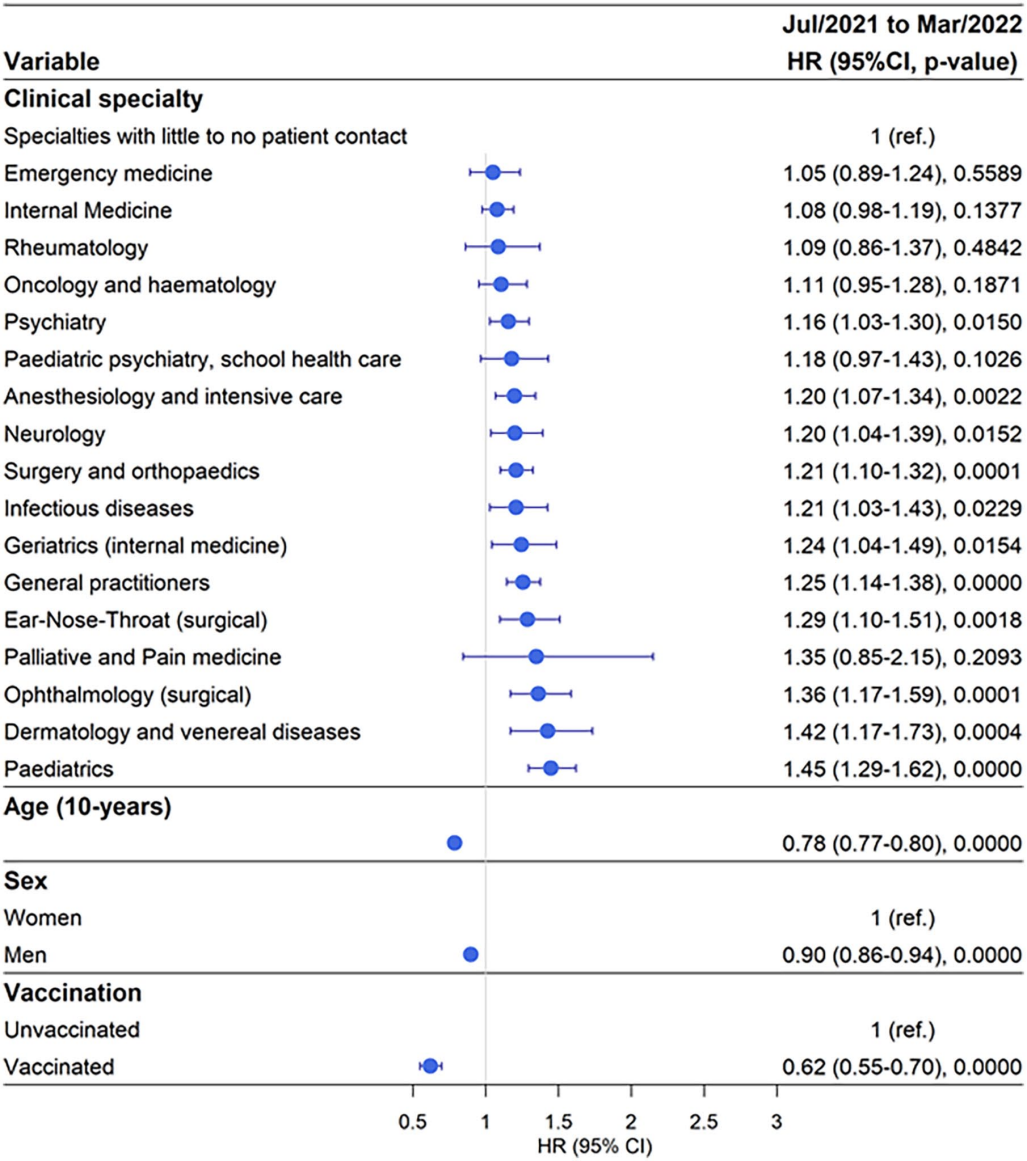
Risk of testing positive for SARS-CoV-2 by clinical speciality adjusted for age, sex, and vaccination status during the time period July 2021 to March 2022. HR: hazard ratio; CI: confidence interval; ref.: reference

Out of 32,654 doctors 7982 (24%) tested positive for SARS-CoV-2, which was the highest proportion of test-positivity compared with the two previous time periods where 12% and 5% tested positive for SARS-CoV-2, respectively. In the reference category 20.36% of doctors tested positive for SARS-CoV-2 (Table I and Supplemental Table SVI). During this period 28.7% of doctors working in paediatric clinics tested positive with aHR 1.45, 95% CI 1.29–1.62; followed by 28.84% of doctors working in Dermatology testing positive with aHR 1.42, 95% CI 1.17–1.73 (Figure 3 and Supplemental Tables SVI and SVII). Furthermore, 27.95% of doctors working in ophthalmology clinics tested positive with aHR 1.36, 95% CI 1.17–1.59. Doctors working in the clinical specialities ENT, General Practice, Geriatrics, Infectious Diseases, Surgery and Orthopaedics, Neurology, Anaesthesiology and Intensive Care, and Psychiatry had a significantly higher risk of testing positive for SARS-CoV-2 compared with the reference group (Figure 3 and Supplemental Tables SVI and SVII). The effect of increasing age remained constant, with 12% decreased risk of testing SARS-CoV-2 positive per decade. In this period, men had 10% decreased risk of testing SARS-CoV-2 positive. The protective effect of vaccination remained at a similar level as during the previous period, with an approximately 40% decreased risk of testing positive for SARS-CoV-2 (Figure 3 and Supplemental Tables SVI and SVII).

## Discussion

We identify doctors working in hospital-based clinics with close contact with contagious patients as carrying the highest occupational-related risk of testing positive for SARS-CoV-2. This is based on unique encompassing data for all publicly employed doctors during 2020–2022 in Sweden, which account for the majority of all doctors in Sweden. Our study spans across both the pre- and the post-vaccine era of the pandemic, which enables us to compare these stages. During the pre-vaccine period, we found the highest risk among doctors working in clinical specialities assessing and caring for the brunt of suspected or confirmed COVID-19 inpatients, infectious disease doctors in particular being at risk. At the time, the Swedish Public Health Agency recommended the general public to limit their social contacts. Thereby, occupational exposure was likely the most significant source of exposure during this period for doctors. During the last period studied in our study, societal transmission was high, thereby rendering it more difficult to determine whether it is occupational or societal transmission leading to positive SARS-CoV-2 testing.

Our findings are supported by studies showing that HCWs have an increased risk of exposure to COVID-19 and thereby also of occupational-related infection, caused by close handling of infectious patients [16,17]. The risk factors for COVID-19 infection among HCWs have been studied in several studies and comprise inadequate personal protective equipment (PPE), poor hand hygiene, involvement in intubations, direct patient contact, or contact with bodily secretions [18–20]. Prior studies have attempted to assess which categories of HCWs are most at risk of COVID-19 infection. A large British study found the highest risks in those working in additional clinical services, nursing and midwifery and in allied health professions [21]. Working in acute medicine, working as a porter or cleaner and on COVID-19 wards during the early phases of the pandemic have been associated with an elevated risk during the early phases of the pandemic [22]. A Finnish nationwide study spanning the first year and a half of the pandemic found that nursing assistants and nurses had the highest risk of COVID-19 among HCWs, especially if they were male and had an immigrant background [23]. A Danish nationwide study found the highest risk of COVID-19 among nursing professionals and specialist medical practitioners [12]. A Norwegian nationwide study covering the first and second pandemic wave, found that nurses, doctors and dentists had the highest risk compared with other professions [24]. In a Swedish nationwide study from the first year of the pandemic, the highest incidence of infection was found among doctors and nurses, closely followed by healthcare assistants [7]. However, many other studies performed lack a nationwide approach, rarely stretch beyond the first pandemic wave and use a crude categorization of HCW. Furthermore, the International Standard of Classification of Occupation codes used in some of the nationwide studies mentioned above lack the sensitivity to identify HCWs according to their clinical work place, and in the case of doctors are so general that they preclude a study similar to ours. Other studies have used seropositivity in HCWs as an indirect measure of past exposure to SARS-CoV-2 [25]. Two studies performed in a Swedish setting during the early stages of the COVID-19 pandemic found highest seropositivity against SARS-CoV-2 for healthcare assistants, followed by nurses and then physicians [26,27]. Presuming that this is transferrable to our results, it would imply that healthcare assistants and nurses working in these clinical specialities would have even higher risks of occupational SARS-CoV-2 infection. However, the study performed by Elfström et al. did not find an increased SARS-CoV-2 seropositivity for infectious disease physicians [27], in contrast to our study. Their study is performed during a shorter time period, utilizes seropositivity and not SARS-CoV-2 tests and the study population is derived from a single centre study setting. In our study we stratify doctors according to clinical work place, incorporating all publicly employed doctors during pre-vaccination and vaccination periods of the COVID-19 pandemic. Different clinical specializations likely have different roles across countries, necessitating knowledge of the country being studied; that is, in Sweden, Infection Control doctors (vårdhygien) in general do not meet with patients, therefore, these were placed in the reference category.

We stratified our analysis into pre-vaccine; vaccine roll-out and post-vaccine time periods, since vaccines reduce the risk of severe COVID-19. In addition, early during the pandemic there were large issues regarding personal protective equipment deliveries, which were resolved during the later phase of the COVID-19 pandemic. These aspects have a large impact on the general risk of occupational-related infection and need to be considered. We initially identified Infection Medicine doctors as those with the highest risk of testing positive for SARS-CoV-2; however, this speciality was among one of the first specialities to be prioritized for vaccines when these were available, which would partially explain why Infection Medicine doctors do not appear to be at increased risk of testing positive for SARS-CoV-2 in the later stages of the pandemic. Finally, in the last phase of the pandemic in our study (ending March 2022), the societal transmission was very high, thereby the proportion of SARS-CoV-2 positive tests attributed to occupational risk is more difficult to interpret.

Physicians working in Paediatric Psychiatry and School Health Care had the highest risk of testing positive for SARS-CoV-2 during the second phase (vaccine roll-out phase). During the early phase of the COVID-19 pandemic, outpatient clinic contacts were decreased and school children received online schooling, thereby minimizing the risk of SARS-CoV-2 transmission. However, during the later phases of the pandemic when children returned to school and outpatient clinics resumed their activities, we speculate that SARS-CoV-2 transmission increased among children, leading to a higher occupational exposure of physicians working with this patient category. In addition, doctors working in dermatology and ophthalmology clinics had the second and third highest risk of testing positive for SARS-CoV-2 in the third phase (post-vaccination phase); their risk had not been significantly increased in the first or second phase. As above, dermatology and ophthalmology clinics decreased or even shut down outpatient contact during the pandemic. When outpatient clinics opened up again, patient contact increased, carrying a higher risk of getting infected with subsequent positive SARS-CoV-2 testing.

We acknowledge limitations to our study. Our study captured all doctors employed in the public sector in Sweden, thus the results may not be representative of doctors working in the private sector, which is a potential limitation. However, the majority of hospital-based clinics are public [14], thereby, our results remain representative for doctors working in these specialities. The private sector is mainly focused on private primary care [14]. The lack of information regarding number of tests and negative tests performed for each individual is a limitation, in that some clinical specialities may have a higher testing frequency compared with other specialities. However, the Swedish Public Health Authorities’ recommendation to test symptomatic HCWs was in place up until April 2022, which is why we limit our study to March 2022 to avoid differential routines for testing across specialities.

The results also need to be interpreted in the context of the progression of the pandemic, keeping in mind factors such as progressive spread through the population in waves, build-up of testing capacity, gradual vaccination roll-out, time elapsed since last vaccine dose, and the arrival of new variants such as Omicron, causing vaccine break-through infections [28]. The more contagious Omicron variant caused break-through infections and reinfections in spite of a >80% prevalence of anti-SARS-CoV-S antibodies in the population, resulting in the highest incidence among the general public during the whole pandemic [2,28]. Thereby, isolating the occupational-related risk during this specific period becomes more difficult. However, we are studying doctors in different clinical specialities that lived through the same societal context, providing a contemporary group of doctors as the reference group. In addition, we do not have access to information regarding PPE usage that each clinic implemented, and which likely changed dynamically across the different phases of the pandemic. Thereby, there are likely time periods where medical doctors could be at higher exposure due to differences in PPE routines.

In conclusion, our findings based on data for all publicly employed doctors in Sweden indicate an increased occupational risk of SARS-CoV-2 infection for doctors handling contagious COVID-19 patients. In future pandemics, interventions and vaccines need to focus on doctors working in these clinical specialities. Further studies are needed to identify where the exposure takes place to inform any preventive interventions.

## Supplemental Material

sj-docx-1-sjp-10.1177_14034948241304487 – Supplemental material for Occupational-related risk of testing SARS-CoV-2 positive for publicly employed medical doctors in Sweden: A nationwide cohort study Supplemental material, sj-docx-1-sjp-10.1177_14034948241304487 for Occupational-related risk of testing SARS-CoV-2 positive for publicly employed medical doctors in Sweden: A nationwide cohort study by Osvaldo Fonseca-Rodriguez, Emma Tobjörk, Hanna Jerndal, Marie Eriksson and Anne-Marie Fors Connolly in Scandinavian Journal of Public Health

sj-pptx-2-sjp-10.1177_14034948241304487 – Supplemental material for Occupational-related risk of testing SARS-CoV-2 positive for publicly employed medical doctors in Sweden: A nationwide cohort study Supplemental material, sj-pptx-2-sjp-10.1177_14034948241304487 for Occupational-related risk of testing SARS-CoV-2 positive for publicly employed medical doctors in Sweden: A nationwide cohort study by Osvaldo Fonseca-Rodriguez, Emma Tobjörk, Hanna Jerndal, Marie Eriksson and Anne-Marie Fors Connolly in Scandinavian Journal of Public Health
