# Supplementary material for: Occupational-related risk of testing SARS-CoV-2 positive for publicly employed medical doctors in Sweden: A nationwide cohort study
Source: Scand J Public Health. 2024 Dec 26;53(5):498–507. doi: 10.1177/14034948241304487 (PMC12159338; doi:10.1177/14034948241304487)
Supplement: sj-docx-1-sjp-10.1177_14034948241304487 – Supplemental material for Occupational-related risk of testing SARS-CoV-2 positive for publicly employed medical doctors in Sweden: A nationwide cohort study [file sj-docx-1-sjp-10.1177_14034948241304487.docx]

**Supplementary material**

**Table S1:** Specialty codes and their corresponding exposure group variable.

| **Specialty codes** | **Exposure categories** |
| --- | --- |
| 80010 | General practitioners |
| 80030 | Infectious diseases |
| 80110 | Emergency medicine |
| 10040  r | Anesthesiology and intensive care |
| 20010, 20020, 20030, 20040, 20050, 20080, 99001 | Internal Medicine |
| 20070 | Geriatrics (internal medicine) |
| 70010, 70020, 70030, 70040 | Neurological and Neurosurgical Specialties |
| 10010, 10020, 10040, 10050, 10080, 10090, 10110, 10120, 10130, 99004, 10030 | Surgery and orthopedics |
| 10070 | Ophthalmology (surgical) |
| 10060, 10061, 10062 | Ear-Nose-Throat (surgical) |
| 30010, 30011, 30012, 30013, 30014, 30015 | Pediatrics |
| 99007, 40020 | Pediatric psychiatry, school health care |
| 40010, 40011, 99003, 99010 | Psychiatry (excl. pediatric psychiatry) |
| 80020, 20060 | Oncology and hematology |
| 80060 | Dermatology and venereal diseases |
| 80070 | Rheumatology |
| 99006, 99008 | Palliative and Pain medicine |
| 60010, 60020, 60030, 60050, 80000, 80040, 80050, 80080, 80090, 99009, 99002, 50010, 50011, 50020, 99005 | Specialties with little to no patient contact (Reference)  Clinical Immunology and Transfusion Medicine; Clinical Microbiology; Clinical Chemistry; Pathology; Clinical Pharmacology; Clinical Genetics; Occupational Medicine; Social Medicine; Forensic Pathology; Clinical Hygiene (vårdhygien); Radiology; Neuroradiology; Clinical Physiology; Nuclear Medicine |
| 99999 | Unknown (exclude) |

**Table S2**: Medical doctors cohort. Period 1 (Feb/2020 to Dec/2020).

|  | COVID-19 | | Sex | | Age | Vaccination | |
| --- | --- | --- | --- | --- | --- | --- | --- |
| Variable | Not positive, N = 28,180^1^ | Positive, N = 3,918^1^ | Women, N = 16,605^1^ | Men, N = 15,493^1^ | N = 32,098^2^ | Unvaccinated, N = 32,019^1^ | Vaccinated, N = 79^1^ |
| **Occupational clinic** |  |  |  |  |  |  |  |
| Specialties with little to no patient contact | 2,796 (9.92%) | 267 (6.81%) | 1,532 (9.23%) | 1,531 (9.88%) | 47 (12) | 3,062 (9.56%) | 1 (1.27%) |
| Anesthesiology and intensive care | 1,846 (6.55%) | 286 (7.30%) | 866 (5.22%) | 1,266 (8.17%) | 46 (11) | 2,131 (6.66%) | 1 (1.27%) |
| Dermatology and venereal diseases | 362 (1.28%) | 38 (0.97%) | 293 (1.76%) | 107 (0.69%) | 44 (11) | 400 (1.25%) | 0 (0.00%) |
| Ear-Nose-Throat (surgical) | 628 (2.23%) | 90 (2.30%) | 350 (2.11%) | 368 (2.38%) | 45 (12) | 718 (2.24%) | 0 (0.00%) |
| Emergency medicine | 590 (2.09%) | 117 (2.99%) | 345 (2.08%) | 362 (2.34%) | 38 (8) | 707 (2.21%) | 0 (0.00%) |
| General practitioners | 5,187 (18.41%) | 670 (17.10%) | 3,299 (19.87%) | 2,558 (16.51%) | 46 (13) | 5,797 (18.10%) | 60 (75.95%) |
| Geriatrics (internal medicine) | 480 (1.70%) | 91 (2.32%) | 372 (2.24%) | 199 (1.28%) | 48 (12) | 567 (1.77%) | 4 (5.06%) |
| Infectious diseases | 573 (2.03%) | 145 (3.70%) | 398 (2.40%) | 320 (2.07%) | 44 (12) | 712 (2.22%) | 6 (7.59%) |
| Internal Medicine | 3,642 (12.92%) | 646 (16.49%) | 1,912 (11.51%) | 2,376 (15.34%) | 45 (12) | 4,287 (13.39%) | 1 (1.27%) |
| Neurology | 841 (2.98%) | 137 (3.50%) | 459 (2.76%) | 519 (3.35%) | 47 (12) | 978 (3.05%) | 0 (0.00%) |
| Oncology and haematology | 861 (3.06%) | 138 (3.52%) | 590 (3.55%) | 409 (2.64%) | 46 (12) | 997 (3.11%) | 2 (2.53%) |
| Ophthalmology (surgical) | 699 (2.48%) | 62 (1.58%) | 448 (2.70%) | 313 (2.02%) | 45 (11) | 761 (2.38%) | 0 (0.00%) |
| Paediatric psychiatry, school health care | 463 (1.64%) | 33 (0.84%) | 394 (2.37%) | 102 (0.66%) | 46 (12) | 496 (1.55%) | 0 (0.00%) |
| Paediatrics | 1,685 (5.98%) | 254 (6.48%) | 1,260 (7.59%) | 679 (4.38%) | 46 (12) | 1,939 (6.06%) | 0 (0.00%) |
| Palliative and Pain medicine | 52 (0.18%) | 4 (0.10%) | 36 (0.22%) | 20 (0.13%) | 52 (8) | 56 (0.17%) | 0 (0.00%) |
| Psychiatry | 1,974 (7.00%) | 182 (4.65%) | 1,137 (6.85%) | 1,019 (6.58%) | 46 (12) | 2,155 (6.73%) | 1 (1.27%) |
| Rheumatology | 298 (1.06%) | 34 (0.87%) | 222 (1.34%) | 110 (0.71%) | 46 (11) | 332 (1.04%) | 0 (0.00%) |
| Surgery and orthopaedics | 5,203 (18.46%) | 724 (18.48%) | 2,692 (16.21%) | 3,235 (20.88%) | 45 (12) | 5,924 (18.50%) | 3 (3.80%) |
| ^1^n (%) | | | | | | | |
| ^2^Age: Mean (SD) | | | | | | | |

**Table S3:** Univariable and multivariable Cox regression models. Period 1: Feb/2020 to Dec/2020

|  | COVID-19 | | Univariable | | Multivariable | |
| --- | --- | --- | --- | --- | --- | --- |
| Variable | Overall, N = 32,098^1^ | Positive, N = 3,918^1^ | HR (95%CI)^2^ | p-value | HR (95%CI)^2^ | p-value |
| **Occupational clinic** |  |  |  |  |  |  |
| Specialties with little to no patient contact | 3,063 | 267 (8.72) | — |  | — |  |
| Paediatric psychiatry, school health care | 496 | 33 (6.65) | 0.75 (0.53, 1.08) | 0.1238 | 0.77 (0.54, 1.11) | 0.1655 |
| Palliative and Pain medicine | 56 | 4 (7.14) | 0.81 (0.30, 2.15) | 0.6682 | 0.88 (0.33, 2.34) | 0.7935 |
| Ophthalmology (surgical) | 761 | 62 (8.15) | 0.93 (0.71, 1.23) | 0.6266 | 0.92 (0.70, 1.22) | 0.5691 |
| Psychiatry | 2,156 | 182 (8.44) | 0.96 (0.80, 1.16) | 0.6995 | 0.96 (0.79, 1.15) | 0.6389 |
| Dermatology and venereal diseases | 400 | 38 (9.50) | 1.09 (0.78, 1.54) | 0.6035 | 1.09 (0.77, 1.53) | 0.6287 |
| Rheumatology | 332 | 34 (10.24) | 1.18 (0.83, 1.69) | 0.3571 | 1.20 (0.84, 1.71) | 0.3189 |
| General practitioners | 5,857 | 670 (11.44) | 1.33 (1.16, 1.53) | 0.0001 | 1.33 (1.16, 1.54) | 0.0001 |
| Surgery and orthopaedics | 5,927 | 724 (12.22) | 1.44 (1.25, 1.65) | 0.0000 | 1.40 (1.22, 1.61) | 0.0000 |
| Ear-Nose-Throat (surgical) | 718 | 90 (12.53) | 1.47 (1.15, 1.86) | 0.0017 | 1.44 (1.13, 1.83) | 0.0028 |
| Paediatrics | 1,939 | 254 (13.10) | 1.55 (1.30, 1.84) | 0.0000 | 1.56 (1.32, 1.86) | 0.0000 |
| Anesthesiology and intensive care | 2,132 | 286 (13.41) | 1.60 (1.35, 1.89) | 0.0000 | 1.56 (1.32, 1.85) | 0.0000 |
| Oncology and haematology | 999 | 138 (13.81) | 1.62 (1.32, 1.99) | 0.0000 | 1.63 (1.33, 2.00) | 0.0000 |
| Neurology | 978 | 137 (14.01) | 1.65 (1.35, 2.03) | 0.0000 | 1.65 (1.34, 2.02) | 0.0000 |
| Internal Medicine | 4,288 | 646 (15.07) | 1.79 (1.55, 2.06) | 0.0000 | 1.74 (1.51, 2.00) | 0.0000 |
| Emergency medicine | 707 | 117 (16.55) | 1.98 (1.60, 2.46) | 0.0000 | 1.79 (1.44, 2.22) | 0.0000 |
| Geriatrics (internal medicine) | 571 | 91 (15.94) | 1.92 (1.51, 2.44) | 0.0000 | 1.98 (1.55, 2.51) | 0.0000 |
| Infectious diseases | 718 | 145 (20.19) | 2.53 (2.06, 3.10) | 0.0000 | 2.48 (2.02, 3.04) | 0.0000 |
| **Age (10 years)** | 45.49 (11.83) | 43.94 (10.92) | 0.88 (0.86, 0.91) | 0.0000 | 0.89 (0.86, 0.91) | 0.0000 |
| **Sex** |  |  |  |  |  |  |
| Women | 16,605 | 1,942 (11.70) | — |  | — |  |
| Men | 15,493 | 1,976 (12.75) | 1.10 (1.03, 1.17) | 0.0039 | 1.11 (1.05, 1.19) | 0.0009 |
| **Vaccination** |  |  |  |  |  |  |
| Unvaccinated | 32,019 | 3,918 (12.24) |  |  |  |  |
| Vaccinated | 79 | 0 (0.00) |  |  |  |  |
| ^1^n (%); Mean (SD) | | | | | | |
| ^2^HR = Hazard Ratio | | | | | | |

**Table S4:** Medical doctors cohort. Period 2 (Jan/2021 to Jun/2021).

|  | COVID-19 | | Sex | | Age | Vaccination | |
| --- | --- | --- | --- | --- | --- | --- | --- |
| Variable | Not positive, N = 30,955^1^ | Positive, N = 1,699^1^ | Women, N = 16,985^1^ | Men, N = 15,669^1^ | N = 32,654^2^ | Unvaccinated, N = 3,716^1^ | Vaccinated, N = 28,938^1^ |
| **Occupational clinic** |  |  |  |  |  |  |  |
| Specialties with little to no patient contact | 2,998 (9.69%) | 145 (8.53%) | 1,601 (9.43%) | 1,542 (9.84%) | 47 (12) | 475 (12.78%) | 2,668 (9.22%) |
| Anesthesiology and intensive care | 2,112 (6.82%) | 77 (4.53%) | 915 (5.39%) | 1,274 (8.13%) | 46 (11) | 137 (3.69%) | 2,052 (7.09%) |
| Dermatology and venereal diseases | 398 (1.29%) | 25 (1.47%) | 307 (1.81%) | 116 (0.74%) | 45 (12) | 56 (1.51%) | 367 (1.27%) |
| Ear-Nose-Throat (surgical) | 689 (2.23%) | 35 (2.06%) | 356 (2.10%) | 368 (2.35%) | 45 (12) | 60 (1.61%) | 664 (2.29%) |
| Emergency medicine | 716 (2.31%) | 29 (1.71%) | 356 (2.10%) | 389 (2.48%) | 38 (8) | 71 (1.91%) | 674 (2.33%) |
| General practitioners | 5,643 (18.23%) | 286 (16.83%) | 3,355 (19.75%) | 2,574 (16.43%) | 47 (13) | 646 (17.38%) | 5,283 (18.26%) |
| Geriatrics (internal medicine) | 551 (1.78%) | 35 (2.06%) | 393 (2.31%) | 193 (1.23%) | 47 (12) | 61 (1.64%) | 525 (1.81%) |
| Infectious diseases | 685 (2.21%) | 36 (2.12%) | 386 (2.27%) | 335 (2.14%) | 45 (12) | 60 (1.61%) | 661 (2.28%) |
| Internal Medicine | 4,106 (13.26%) | 245 (14.42%) | 1,932 (11.37%) | 2,419 (15.44%) | 45 (12) | 490 (13.19%) | 3,861 (13.34%) |
| Neurology | 978 (3.16%) | 38 (2.24%) | 487 (2.87%) | 529 (3.38%) | 47 (12) | 105 (2.83%) | 911 (3.15%) |
| Oncology and haematology | 986 (3.19%) | 41 (2.41%) | 609 (3.59%) | 418 (2.67%) | 46 (12) | 100 (2.69%) | 927 (3.20%) |
| Ophthalmology (surgical) | 737 (2.38%) | 43 (2.53%) | 446 (2.63%) | 334 (2.13%) | 45 (11) | 104 (2.80%) | 676 (2.34%) |
| Paediatric psychiatry, school health care | 472 (1.52%) | 35 (2.06%) | 406 (2.39%) | 101 (0.64%) | 46 (11) | 86 (2.31%) | 421 (1.45%) |
| Paediatrics | 1,856 (6.00%) | 128 (7.53%) | 1,294 (7.62%) | 690 (4.40%) | 46 (12) | 236 (6.35%) | 1,748 (6.04%) |
| Palliative and Pain medicine | 74 (0.24%) | 4 (0.24%) | 51 (0.30%) | 27 (0.17%) | 54 (8) | 7 (0.19%) | 71 (0.25%) |
| Psychiatry | 2,054 (6.64%) | 121 (7.12%) | 1,140 (6.71%) | 1,035 (6.61%) | 46 (12) | 370 (9.96%) | 1,805 (6.24%) |
| Rheumatology | 321 (1.04%) | 19 (1.12%) | 231 (1.36%) | 109 (0.70%) | 47 (12) | 38 (1.02%) | 302 (1.04%) |
| Surgery and orthopaedics | 5,579 (18.02%) | 357 (21.01%) | 2,720 (16.01%) | 3,216 (20.52%) | 45 (12) | 614 (16.52%) | 5,322 (18.39%) |
| ^1^n (%) | | | | | | | |
| ^2^Age: Mean (SD) | | | | | | | |

**Table S5:** Univariable and multivariable Cox regression models. Period 2: Jan/2021 to June/2021

|  | COVID-19 | | Univariable | | Multivariable | |
| --- | --- | --- | --- | --- | --- | --- |
| Variable | Overall, N = 32,654^1^ | Positive, N = 1,699^1^ | HR (95%CI)^2^ | p-value | HR (95%CI)^2^ | p-value |
| **Occupational clinic** |  |  |  |  |  |  |
| Specialties with little to no patient contact | 3,143 | 145 (4.61) | — |  | — |  |
| Neurology | 1,016 | 38 (3.74) | 0.81 (0.57, 1.15) | 0.2424 | 0.85 (0.59, 1.21) | 0.3590 |
| Anesthesiology and intensive care | 2,189 | 77 (3.52) | 0.76 (0.58, 1.00) | 0.0537 | 0.89 (0.68, 1.18) | 0.4365 |
| Emergency medicine | 745 | 29 (3.89) | 0.85 (0.57, 1.26) | 0.4138 | 0.90 (0.60, 1.35) | 0.6060 |
| Oncology and haematology | 1,027 | 41 (3.99) | 0.87 (0.61, 1.23) | 0.4270 | 0.92 (0.65, 1.31) | 0.6483 |
| Ear-Nose-Throat (surgical) | 724 | 35 (4.83) | 1.06 (0.73, 1.53) | 0.7704 | 1.14 (0.78, 1.65) | 0.4980 |
| General practitioners | 5,929 | 286 (4.82) | 1.05 (0.86, 1.28) | 0.6213 | 1.15 (0.94, 1.40) | 0.1764 |
| Ophthalmology (surgical) | 780 | 43 (5.51) | 1.20 (0.86, 1.69) | 0.2825 | 1.20 (0.86, 1.69) | 0.2872 |
| Psychiatry | 2,175 | 121 (5.56) | 1.21 (0.95, 1.55) | 0.1131 | 1.21 (0.95, 1.54) | 0.1165 |
| Infectious diseases | 721 | 36 (4.99) | 1.09 (0.76, 1.58) | 0.6305 | 1.23 (0.85, 1.78) | 0.2668 |
| Rheumatology | 340 | 19 (5.59) | 1.22 (0.76, 1.97) | 0.4117 | 1.28 (0.80, 2.07) | 0.3056 |
| Dermatology and venereal diseases | 423 | 25 (5.91) | 1.30 (0.85, 1.98) | 0.2316 | 1.29 (0.85, 1.98) | 0.2361 |
| Internal Medicine | 4,351 | 245 (5.63) | 1.24 (1.01, 1.52) | 0.0412 | 1.32 (1.08, 1.63) | 0.0076 |
| Palliative and Pain medicine | 78 | 4 (5.13) | 1.15 (0.41, 3.17) | 0.7923 | 1.36 (0.49, 3.77) | 0.5505 |
| Surgery and orthopaedics | 5,936 | 357 (6.01) | 1.32 (1.09, 1.60) | 0.0050 | 1.38 (1.14, 1.67) | 0.0011 |
| Geriatrics (internal medicine) | 586 | 35 (5.97) | 1.32 (0.91, 1.91) | 0.1424 | 1.47 (1.02, 2.14) | 0.0415 |
| Paediatrics | 1,984 | 128 (6.45) | 1.41 (1.11, 1.78) | 0.0045 | 1.49 (1.17, 1.88) | 0.0011 |
| Paediatric psychiatry, school health care | 507 | 35 (6.90) | 1.51 (1.05, 2.18) | 0.0272 | 1.50 (1.04, 2.17) | 0.0311 |
| **Age (10 years)** | 45.66 (11.97) | 44.05 (10.63) | 0.89 (0.85, 0.92) | 0.0000 | 0.89 (0.86, 0.93) | 0.0000 |
| **Sex** |  |  |  |  |  |  |
| Women | 16,985 | 901 (5.30) | — |  | — |  |
| Men | 15,669 | 798 (5.09) | 0.96 (0.87, 1.06) | 0.4125 | 1.04 (0.94, 1.14) | 0.4782 |
| **Vaccination** |  |  |  |  |  |  |
| Unvaccinated | 3,716 | 1,314 (35.36) | — |  | — |  |
| Vaccinated | 28,938 | 385 (1.33) | 0.58 (0.51, 0.66) | 0.0000 | 0.60 (0.53, 0.68) | 0.0000 |
| ^1^n (%); Mean (SD) | | | | | | |
| ^2^HR = Hazard Ratio | | | | | | |

**Table S6:** Medical doctors cohort. Period 2 (Jul/2021 to Mar/2022)

|  | COVID-19 | | Sex | | Age | Vaccination | |
| --- | --- | --- | --- | --- | --- | --- | --- |
| Variable | Not positive, N = 24,673^1^ | Positive, N = 7,981^1^ | Women, N = 16,985^1^ | Men, N = 15,669^1^ | N = 32,654^2^ | Unvaccinated, N = 977^1^ | Vaccinated, N = 31,677^1^ |
| **Occupational clinic** |  |  |  |  |  |  |  |
| Specialties with little to no patient contact | 2,503 (10.14%) | 640 (8.02%) | 1,601 (9.43%) | 1,542 (9.84%) | 47 (12) | 152 (15.56%) | 2,991 (9.44%) |
| Anesthesiology and intensive care | 1,656 (6.71%) | 533 (6.68%) | 915 (5.39%) | 1,274 (8.13%) | 46 (11) | 37 (3.79%) | 2,152 (6.79%) |
| Dermatology and venereal diseases | 301 (1.22%) | 122 (1.53%) | 307 (1.81%) | 116 (0.74%) | 45 (12) | 14 (1.43%) | 409 (1.29%) |
| Ear-Nose-Throat (surgical) | 530 (2.15%) | 194 (2.43%) | 356 (2.10%) | 368 (2.35%) | 45 (12) | 10 (1.02%) | 714 (2.25%) |
| Emergency medicine | 554 (2.25%) | 191 (2.39%) | 356 (2.10%) | 389 (2.48%) | 38 (8) | 10 (1.02%) | 735 (2.32%) |
| General practitioners | 4,425 (17.93%) | 1,504 (18.84%) | 3,355 (19.75%) | 2,574 (16.43%) | 47 (13) | 179 (18.32%) | 5,750 (18.15%) |
| Geriatrics (internal medicine) | 438 (1.78%) | 148 (1.85%) | 393 (2.31%) | 193 (1.23%) | 47 (12) | 16 (1.64%) | 570 (1.80%) |
| Infectious diseases | 537 (2.18%) | 184 (2.31%) | 386 (2.27%) | 335 (2.14%) | 45 (12) | 4 (0.41%) | 717 (2.26%) |
| Internal Medicine | 3,354 (13.59%) | 997 (12.49%) | 1,932 (11.37%) | 2,419 (15.44%) | 45 (12) | 126 (12.90%) | 4,225 (13.34%) |
| Neurology | 773 (3.13%) | 243 (3.04%) | 487 (2.87%) | 529 (3.38%) | 47 (12) | 36 (3.68%) | 980 (3.09%) |
| Oncology and haematology | 792 (3.21%) | 235 (2.94%) | 609 (3.59%) | 418 (2.67%) | 46 (12) | 26 (2.66%) | 1,001 (3.16%) |
| Ophthalmology (surgical) | 562 (2.28%) | 218 (2.73%) | 446 (2.63%) | 334 (2.13%) | 45 (11) | 39 (3.99%) | 741 (2.34%) |
| Paediatric psychiatry, school health care | 386 (1.56%) | 121 (1.52%) | 406 (2.39%) | 101 (0.64%) | 46 (11) | 17 (1.74%) | 490 (1.55%) |
| Paediatrics | 1,414 (5.73%) | 570 (7.14%) | 1,294 (7.62%) | 690 (4.40%) | 46 (12) | 42 (4.30%) | 1,942 (6.13%) |
| Palliative and Pain medicine | 60 (0.24%) | 18 (0.23%) | 51 (0.30%) | 27 (0.17%) | 54 (8) | 2 (0.20%) | 76 (0.24%) |
| Psychiatry | 1,663 (6.74%) | 512 (6.42%) | 1,140 (6.71%) | 1,035 (6.61%) | 46 (12) | 137 (14.02%) | 2,038 (6.43%) |
| Rheumatology | 263 (1.07%) | 77 (0.96%) | 231 (1.36%) | 109 (0.70%) | 47 (12) | 13 (1.33%) | 327 (1.03%) |
| Surgery and orthopaedics | 4,462 (18.08%) | 1,474 (18.47%) | 2,720 (16.01%) | 3,216 (20.52%) | 45 (12) | 117 (11.98%) | 5,819 (18.37%) |
| ^1^n (%) | | | | | | | |
| ^2^Age: Mean (SD) | | | | | | | |

**Table S7:** Univariable and multivariable Cox regression models. Period 3: Jul/2021 to Mar/2022

|  | COVID-19 | | Univariable | | Multivariable | |
| --- | --- | --- | --- | --- | --- | --- |
| Variable | Overall, N = 32,654^1^ | Positive, N = 7,981^1^ | HR (95%CI)^2^ | p-value | HR (95%CI)^2^ | p-value |
| **Occupational clinic** |  |  |  |  |  |  |
| Specialties with little to no patient contact | 3,143 | 640 (20.36) | — |  | — |  |
| Emergency medicine | 745 | 191 (25.64) | 1.30 (1.11, 1.53) | 0.0014 | 1.05 (0.89, 1.24) | 0.5589 |
| Internal Medicine | 4,351 | 997 (22.91) | 1.14 (1.03, 1.26) | 0.0083 | 1.08 (0.98, 1.19) | 0.1377 |
| Rheumatology | 340 | 77 (22.65) | 1.13 (0.89, 1.42) | 0.3251 | 1.09 (0.86, 1.37) | 0.4842 |
| Oncology and haematology | 1,027 | 235 (22.88) | 1.14 (0.98, 1.32) | 0.0885 | 1.11 (0.95, 1.28) | 0.1871 |
| Psychiatry | 2,175 | 512 (23.54) | 1.18 (1.05, 1.33) | 0.0044 | 1.16 (1.03, 1.30) | 0.0150 |
| Paediatric psychiatry, school health care | 507 | 121 (23.87) | 1.21 (1.00, 1.47) | 0.0558 | 1.18 (0.97, 1.43) | 0.1026 |
| Anesthesiology and intensive care | 2,189 | 533 (24.35) | 1.23 (1.09, 1.38) | 0.0005 | 1.20 (1.07, 1.34) | 0.0022 |
| Neurology | 1,016 | 243 (23.92) | 1.21 (1.04, 1.40) | 0.0124 | 1.20 (1.04, 1.39) | 0.0152 |
| Surgery and orthopaedics | 5,936 | 1,474 (24.83) | 1.26 (1.15, 1.39) | 0.0000 | 1.21 (1.10, 1.32) | 0.0001 |
| Infectious diseases | 721 | 184 (25.52) | 1.29 (1.10, 1.52) | 0.0022 | 1.21 (1.03, 1.43) | 0.0229 |
| Geriatrics (internal medicine) | 586 | 148 (25.26) | 1.27 (1.06, 1.51) | 0.0086 | 1.24 (1.04, 1.49) | 0.0154 |
| General practitioners | 5,929 | 1,504 (25.37) | 1.29 (1.17, 1.41) | 0.0000 | 1.25 (1.14, 1.38) | 0.0000 |
| Ear-Nose-Throat (surgical) | 724 | 194 (26.80) | 1.36 (1.16, 1.59) | 0.0002 | 1.29 (1.10, 1.51) | 0.0018 |
| Palliative and Pain medicine | 78 | 18 (23.08) | 1.15 (0.72, 1.83) | 0.5612 | 1.35 (0.85, 2.15) | 0.2093 |
| Ophthalmology (surgical) | 780 | 218 (27.95) | 1.43 (1.23, 1.67) | 0.0000 | 1.36 (1.17, 1.59) | 0.0001 |
| Dermatology and venereal diseases | 423 | 122 (28.84) | 1.52 (1.25, 1.85) | 0.0000 | 1.42 (1.17, 1.73) | 0.0004 |
| Paediatrics | 1,984 | 570 (28.73) | 1.49 (1.33, 1.67) | 0.0000 | 1.45 (1.29, 1.62) | 0.0000 |
| **Age (10 years)** | 45.66 (11.97) | 42.86 (9.94) | 0.79 (0.78, 0.81) | 0.0000 | 0.78 (0.77, 0.80) | 0.0000 |
| **Sex** |  |  |  |  |  |  |
| Women | 16,985 | 4,440 (26.14) | — |  | — |  |
| Men | 15,669 | 3,541 (22.60) | 0.84 (0.81, 0.88) | 0.0000 | 0.90 (0.86, 0.94) | 0.0000 |
| **Vaccination** |  |  |  |  |  |  |
| Unvaccinated | 977 | 236 (24.16) | — |  | — |  |
| Vaccinated | 31,677 | 7,745 (24.45) | 0.74 (0.66, 0.83) | 0.0000 | 0.62 (0.55, 0.70) | 0.0000 |
| ^1^n (%); Mean (SD) | | | | | | |
| ^2^HR = Hazard Ratio | | | | | | |
