# Supplementary material for: Occupational-related risk of testing SARS-CoV-2 positive for publicly employed medical doctors in Sweden: A nationwide cohort study
Source: Scand J Public Health. 2024 Dec 26;53(5):498–507. doi: 10.1177/14034948241304487 (PMC12159338; doi:10.1177/14034948241304487)
Supplement: sj-pptx-2-sjp-10.1177_14034948241304487 – Supplemental material for Occupational-related risk of testing SARS-CoV-2 positive for publicly employed medical doctors in Sweden: A nationwide cohort study [file sj-pptx-2-sjp-10.1177_14034948241304487.pptx]

## Slide 1
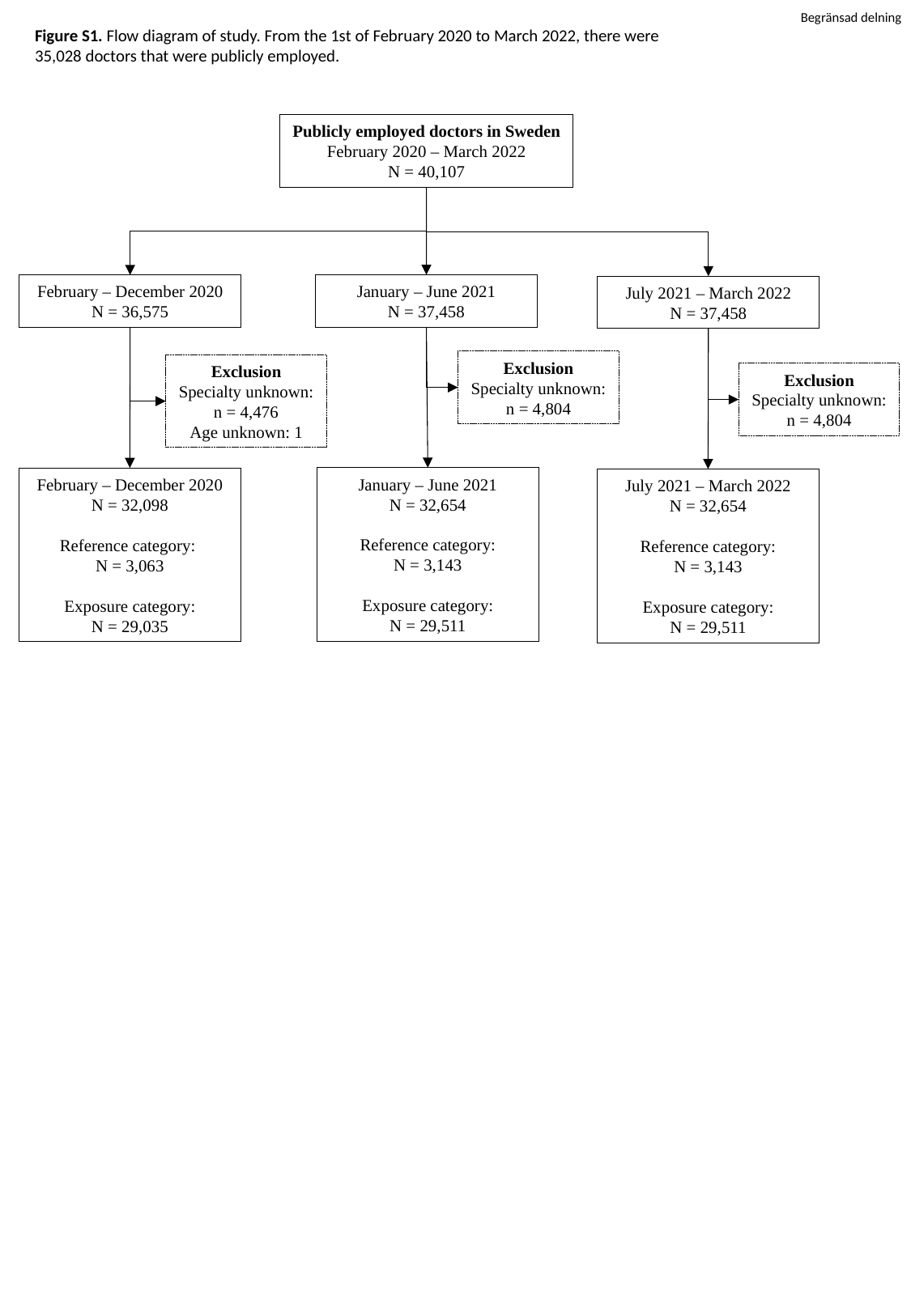

Figure S1. Flow diagram of study. From the 1st of February 2020 to March 2022, there were 35,028 doctors that were publicly employed.
Publicly employed doctors in Sweden February 2020 – March 2022
N = 40,107
January – June 2021
N = 37,458
February – December 2020
N = 36,575
July 2021 – March 2022
N = 37,458
Exclusion
Specialty unknown: n = 4,804
Exclusion
Specialty unknown: n = 4,476
Age unknown: 1
Exclusion
Specialty unknown: n = 4,804
January – June 2021
N = 32,654
Reference category:
N = 3,143
Exposure category:
N = 29,511
February – December 2020
N = 32,098
Reference category:
N = 3,063
Exposure category:
N = 29,035
July 2021 – March 2022
N = 32,654
Reference category:
N = 3,143
Exposure category:
N = 29,511

## Slide 2
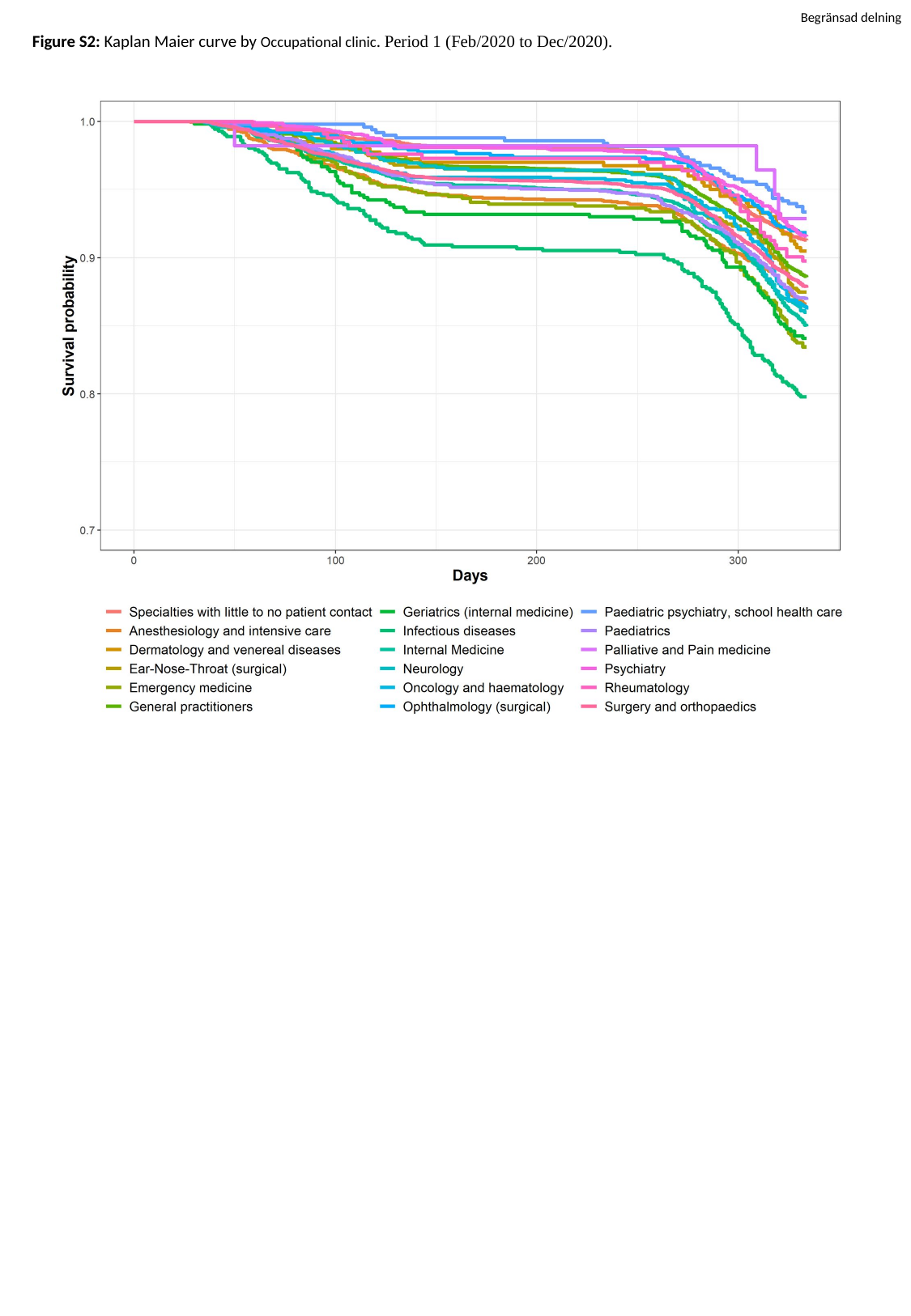

Figure S2: Kaplan Maier curve by Occupational clinic. Period 1 (Feb/2020 to Dec/2020).

## Slide 3
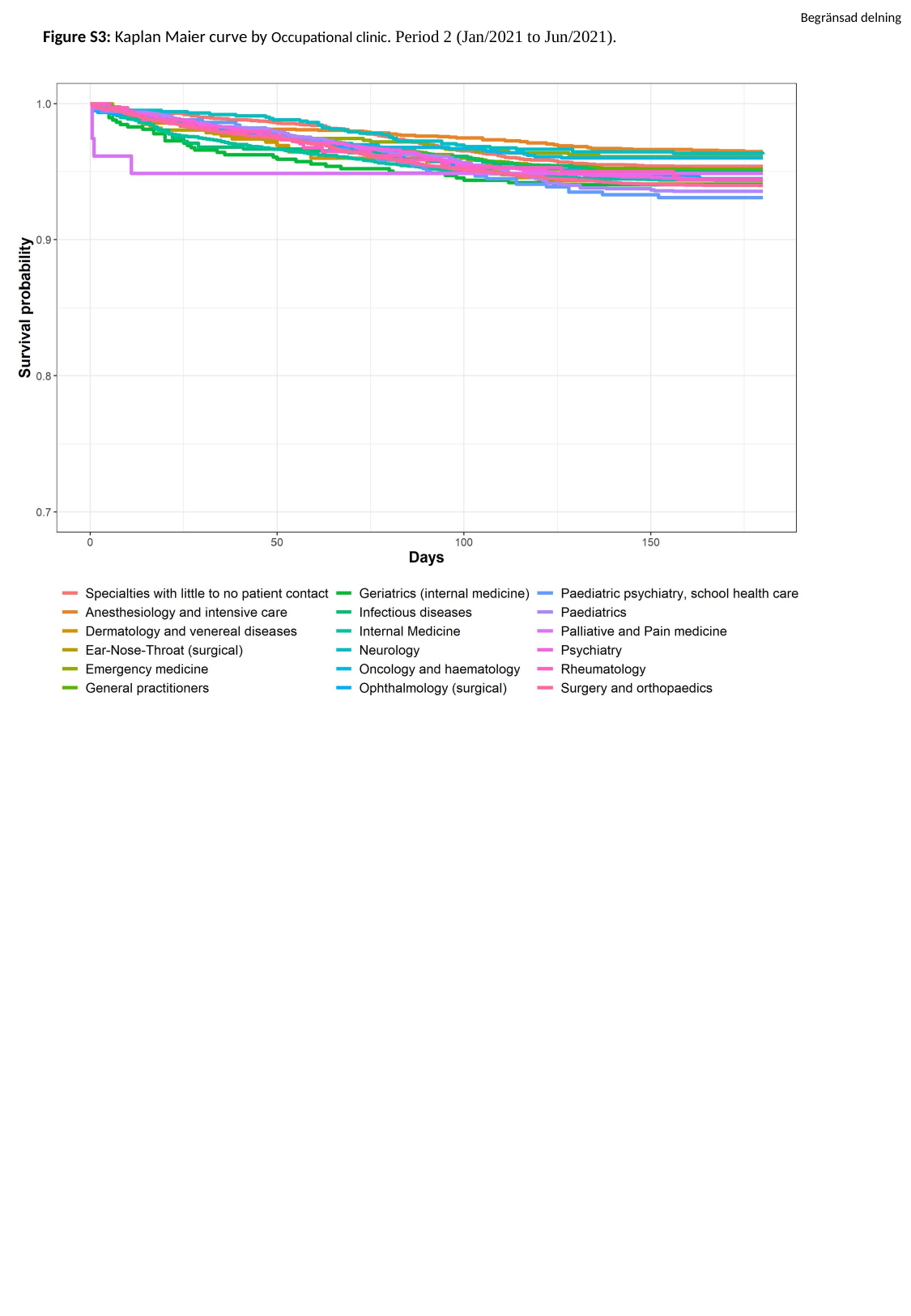

Figure S3: Kaplan Maier curve by Occupational clinic. Period 2 (Jan/2021 to Jun/2021).

## Slide 4
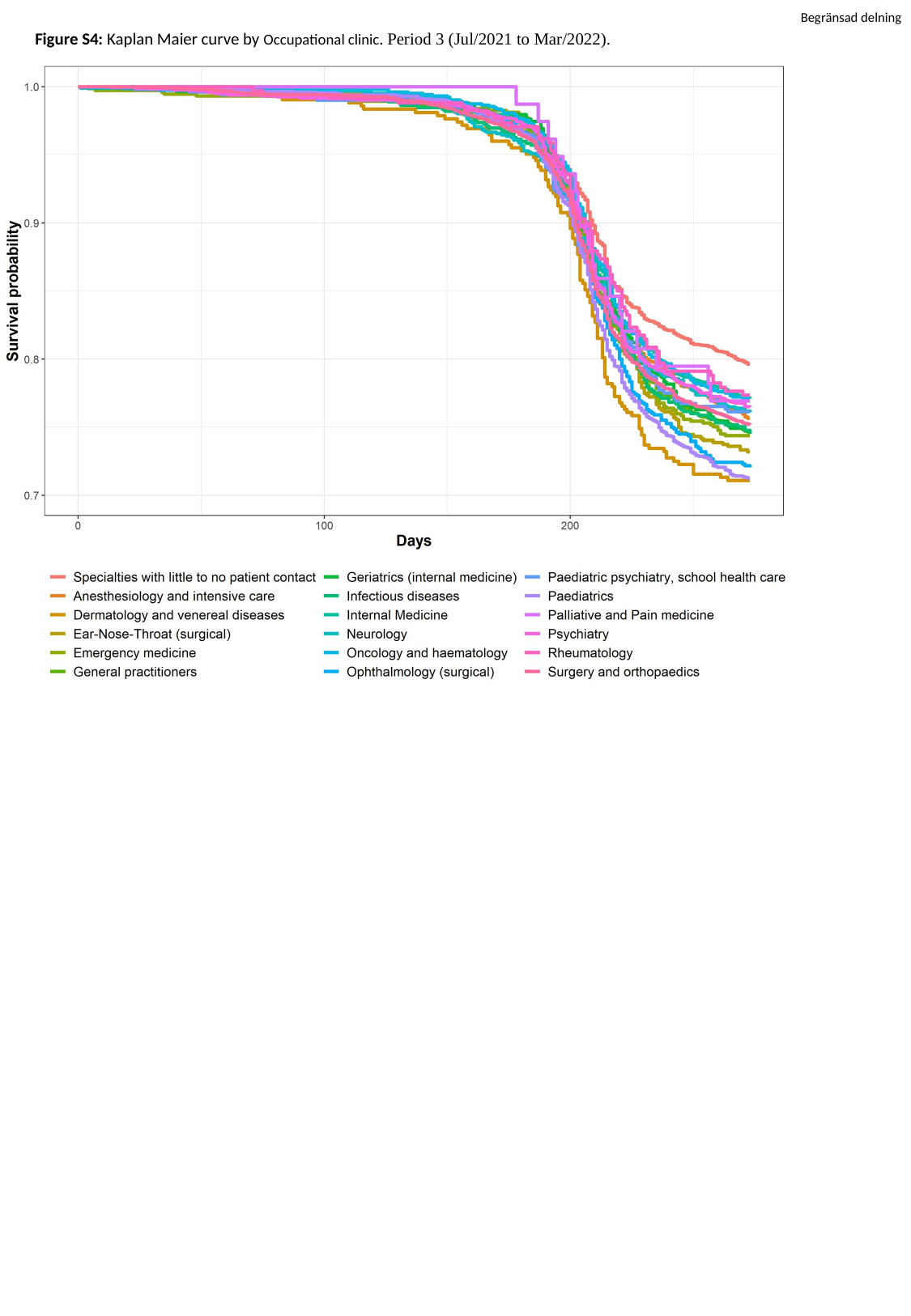

Figure S4: Kaplan Maier curve by Occupational clinic. Period 3 (Jul/2021 to Mar/2022).

## Slide 5
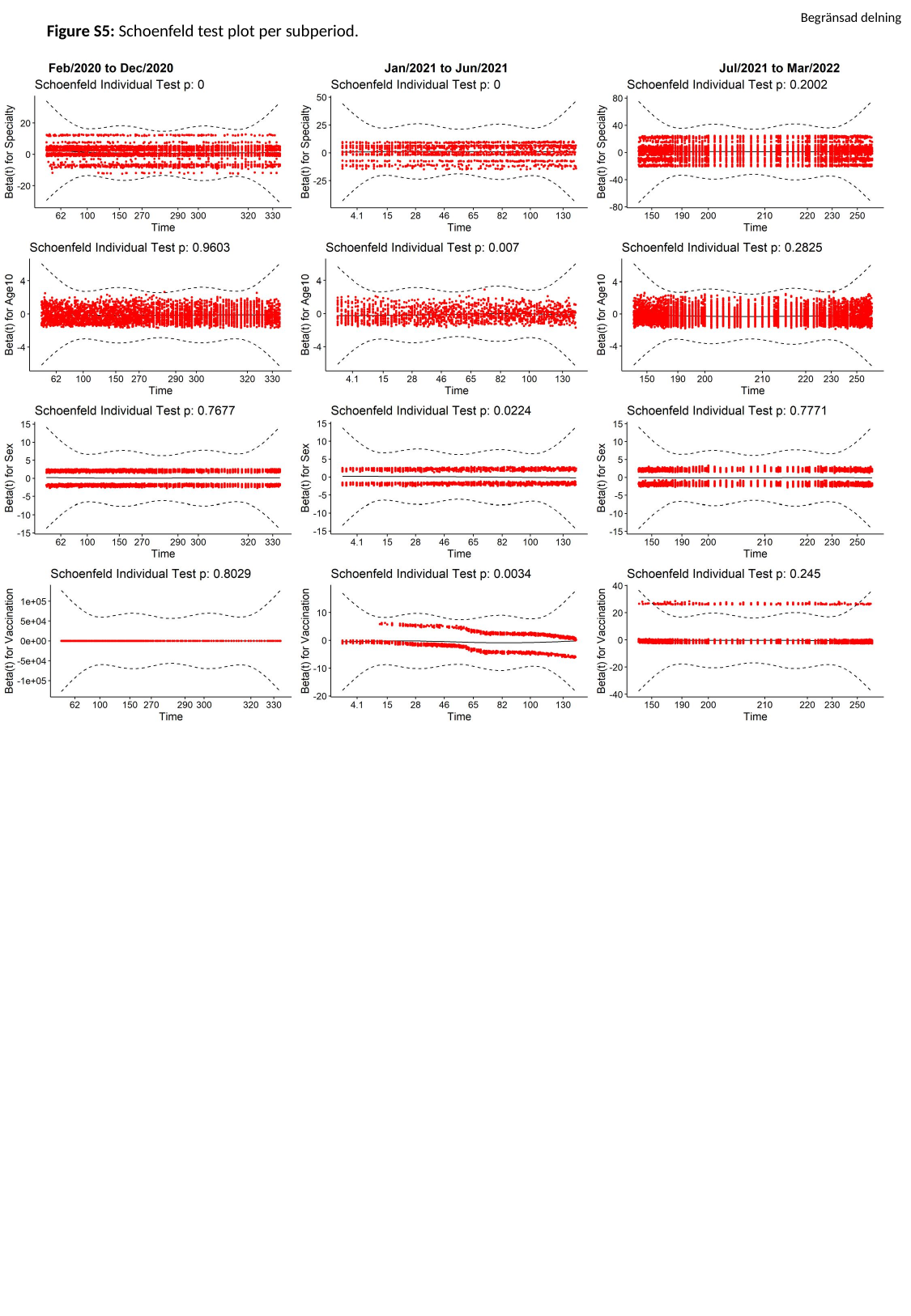

Figure S5: Schoenfeld test plot per subperiod.
